# Effectiveness of a Community-Based Muscle Strengthening Exercise Program to Increase Muscle Strength Among Pre-frail Older Persons in Malaysia: A Pilot Study

**DOI:** 10.3389/fpubh.2021.610184

**Published:** 2021-04-21

**Authors:** Raja Nurzatul Efah Raja Adnan, Hazwan Mat Din, Asmidawati Ashari, Halimatus Sakdiah Minhat

**Affiliations:** ^1^Medical Gerontology Laboratory, Malaysian Research Institute on Ageing, Universiti Putra Malaysia, Serdang, Malaysia; ^2^Department of Human Development and Family Studies, Faculty of Human Ecology, Universiti Putra Malaysia, Serdang, Malaysia; ^3^Department of Community Health, Faculty of Medicine and Health Sciences, Universiti Putra Malaysia, Serdang, Malaysia

**Keywords:** exercise, muscle strength, older persons, community-dwelling, pre-frail

## Abstract

Deterioration in muscle mass and muscle strength is common among the frail older persons, cause functional dependence and decrease in the quality of life. Therefore, the identification of cost-effective interventions to prevent or ameliorate frailty is crucially needed. The aim of this study is to determine the effectiveness of a Community-based Muscle Strengthening Exercise (COME) program to increase muscle strength among pre-frail older persons. This study was a quasi-experimental study. A total of 32 older persons aged 60 years and older with pre-frail symptoms were recruited from the community center. The COME was developed based on the Growing Stronger program and the Otago Home Exercise Program. COME was designed to strengthen all of the major muscle groups in the upper and lower extremities. The exercise program was conducted for 12 weeks and divided into 3 parts; (1) to strengthen the body slowly and gently, using only body weight, (2) to introduce dumbbells and ankle weights to increase strength and (3) to add various new ways to boost strength even more. Functional tests were measured before and after the intervention. The results revealed non-significant *p*-value for pre- and post-intervention comparison for all study outcomes. Observing the values of mean difference, the study intervention was shown to have slightly improved the time up and go (Mean difference = −0.25), and sit-to-stand duration (Mean difference = −0.41) as well as the handgrip strength (Mean difference = 0.68) among the participants. On the assessment of Cohen ES, all three improvements exhibited small effect sizes. Sit-to-stand duration was shown to have most benefited from the intervention with highest ES among the outcome variables (ES = 0.20). COME intervention program among pre-frail older persons showed favorable trend toward improvement of upper and lower extremities muscle strength. This study should be further tested in randomized control trial to confirm its effectiveness.

## Introduction

Frailty is a condition where deficits accumulated and causing vulnerability, which increases the risk of adverse outcomes including falls, delirium, and disability (1). Geriatric frailty is a major health condition associated with aging and has drawn growing attention in recent years due to its associations with multiple adverse outcomes. Deterioration in muscle mass and muscle strength are common among frail older persons and have effects on functional dependence and decrease in quality of life. Therefore, the identification of cost-effective interventions to prevent frailty is critically needed and has become one of the most important public health concerns.

Evidence based studies indicate that progressive strengthening exercise programs have been consistently effective in improving muscle strength and functional ability in older adults, as well as a reduction in the symptoms of various chronic diseases such as arthritis, depression, type II diabetes, osteoporosis, sleep disorders, and cardiovascular disease (2–4). As reported by Cochrane review, multi-component group exercise, including resistance and balance training, reduced the rate of falls by 22% and fall risks by 17% in adults aged 60 years and over (5). Other study found that dynamic standing balance exercise performed at least three times a week over a 3- month period significantly improved balance and mobility among older adults (6). Additionally, regular physical exercise training have been proposed as preventive measures for frailty and its adverse outcomes, targeted most of the frailty criteria such as weakness, low physical activity, slowed motor performance, and exercise intolerance (7). Cadore at al. reviewed several studies of the effects of exercise intervention on muscle strength and most of them showed an increase in muscle strength (8). Although various interventions have been developed to improve the outcomes of frail older people, a major obstacle that impede success in such interventions was due to the differences in the diagnosis of frailty by researchers.

The increasing trend of older population in Malaysia has been linked with increasing proportion of older persons with frailty. A study conducted in an urban area among 473 older persons aged 60 years and above in Klang Valley reported that 61.7% of the respondents were pre-frail and 8.9% were frail elders, which were detected using Fried's criteria (9). Another study on prevalence of frailty among older population aged 60 years and above in East Coast Malaysia revealed that prevalence of frailty syndrome was 18.3% (10). Several exercise intervention studies including multicomponent exercise with lifestyle intervention, and chair-based exercise have been conducted among the older population in Malaysia (11–13). These studies were carried out either among older adults with low resources settings, institutionalized, or at risk fallers to improve physical performance and reduce risk of falls. However, there is lack of intervention studies targeting pre-frail older persons in the community. It is important to strengthen the muscle at pre-frail stage to prevent further loss of muscle strength and minimize the adverse effects of frailty syndrome. Thus, this study aimed to determine the effectiveness of the Community-based Muscle Strengthening Exercise (COME) Program to increase the muscle strength among pre-frail older persons in order to prevent frailty.

## Methods

### Study Design

This study was quasi experimental study with the same group involve as the control and intervention groups.

### Setting and Participants

The recruits included 36 pre-frail older persons aged 60 years and above who are attending the primary health clinic in Seri Petaling District with a Frailty Index Score of 1–2. The Frailty Index score was measured based on the following criteria as proposed by Fried et al. (14); unintended weight loss (5 kgs and more in the past year), self-reported exhaustion (identified by two questions from the CES-D scale), weakness (hand grip is less than the cut-off points mentioned on the original reference, adjusted for gender and body mass index), slow walking speed (walking speed more than the cut-off points mentioned on the original reference, adjusted for gender and height), and low physical activity (<383 kcals per week for men and <270 kcals per week for women). Score 0 indicated non-frail, 1 to 2 indicated pre-frail while 3 and more indicated frail. Other inclusion criteria are older persons with normal blood pressure, absence of abnormal heart sounds and murmurs, clear lungs bilaterally with no added sounds during inspiration and expiration, under control comorbidities, and no history of previous cardiac or respiratory problems in the past 2 weeks to 1 month prior to enrolment into the study. Screening of eligible participants was done by a physician.

### COME Program

The COME program was developed based on the Growing Stronger program proposed by Centers for Disease Control and Prevention, U.S. Department of Health and Human Services (15) and the Otago Home Exercise Program (16). The Growing Stronger program/ book was developed for the older adult who wants to grow stronger, healthier, more active, and more independent. It gives a safe, simple, and highly effective exercise program based on the principles of strength training. Meanwhile, the Otago exercise program was designed specifically to prevent falls which consists of a set of leg muscle strengthening, balance retraining exercises and a walking plan. The program was developed and tested in four randomized controlled trials and one controlled trial. However, the effectiveness of combination of these programs to improve muscle strength specifically among pre-frail older persons is yet to be tested.

COME was designed to strengthen all of the major muscle groups in the upper extremities (shoulders, upper arms, back, chest, and abdomen) and in the lower extremities (hips, thighs, knees, lower legs, and ankles). It also mainly targeted the shoulders, hands, hips, and knees, the muscles usually affected by osteoarthritis. The intervention was in the form of a group exercise program that was conducted by trained instructor for a duration of 12 weeks and consisted of three parts. Part 1 focused on strengthening the body slowly and gently, using only their own body weight while Part 2 introduced weights to increase strength. A variety with new way of exercise was added in Part 3 to further boost strength (Table 1). The instructors experienced training prior to the implementation of the intervention, which was given by a certified physiotherapist. The participants were divided into three groups with 10–14 participants each group according to their location. An instructor was assigned to each group to guide and monitor the participants. The intervention was conducted twice a week at the community hall and their attendance were recorded. Participants who attended at least 20 out of 24 sessions were included in the analysis. At the end of the intervention, the participants were given honorarium as token of appreciation.

**Table 1:** Detailed exercise for part 1, part 2 and part 3 of COME program.

**Part 1 (2 weeks)**	**Part 2 (4 weeks)**	**Part 3 (6 weeks)**
**Warm up: 5-min walk outdoor/ indoor**.
i Squats – 10 reps ii Push-ups: Conducted using terra band, 10 reps iii Toe stands – 10 s, 10 reps. iv Marching – standing or sitting for 30 reps with 1 min rest in between.	i Biceps curl – 3 sets of 10 reps, using 1-pound weight dumbbells with rest in between each set for 1 min. ii Step-ups – number of steps counted for a duration of 1 min. iii Overhead pressure −3 sets of 10 reps using 1-pound weight dumbbells with rest in between each set for 1 min. iv Side hip raise – 10 reps for each leg.	i Knee extension – extend and hold for 10 s, 10 reps for each leg. ii Knee curl – curl and hold for 10 s x 10 reps for each leg. iii Pelvic tilt – hold for 10 s, 10 reps. iv Bridging – hold for 10 s, 10 reps.
**Cool-down: Conducted at the end of every session**.
i Chest & arm stretch ii Hamstring/ calf stretch iii Quadriceps stretch iv Neck, upper back and shoulder stretch		

### Measurements

Pre measurements were taken at the baseline whereby post-intervention measurements were taken after week 12 of the intervention. Socio-demographic characteristics and co-morbidity were assessed at the pre-intervention stage only using self-reported, structured questionnaire. The functional tests including timed up and go, Berg balance scale, sit-to-stand and hand grip strength were assessed before and after the intervention.

#### Timed Up and Go

TUG is a commonly used as screening tool for mobility both in the community and in the inpatient setting. The subject was timed while they rise from an arm chair (approximate seat height 46 cm), walked three meters, turned and walked back to the chair and sat down again (17). The subject practiced the test once before being timed for familiarity. The subject wore his or her regular footwear and used their customary walking aid if necessary. A faster time (in seconds) indicated a better functional mobility performance.

#### Berg Balance Scale

The scale is a performance-oriented measure of balance in older persons. Berg balance scale is a reliable, valid and widely used tool to measure balance. It consisted of 14 items that are scored based on a scale of 0–4 (18). The subject was given 0 score if he/she was unable to do the task, and a score of 4 was given if the subject was able to complete the task based on the assigned criterion. The maximum total score of the test is 56. The items included simple mobility tasks (e.g., transfers, standing unsupported, sit-to-stand) and more difficult tasks (e.g., tandem standing, turning 360°, single-leg stance).

#### Sit-to-Stand

This test has often been seen as an indicator or proxy measure for lower extremity strength in older people. The subject was timed (in seconds) as they stood up and sat down as quickly as possible on a firm, padded, armless chair for five cycles (19). Subject performed the trial twice. The beginning of the timed test was prefaced with, “Ready, Set, Go” by the assessor. The time was recorded after the word “Go,” and the assessor counted aloud each of the five completed cycles. The time recorded was ended when the subject returned to the seated position for the fifth time.

#### Hand Grip Strength

Hand grip strength is a measure of maximum force generated by forearm muscle. A Jamar dynamometer was used to assess hand grip strength. Subject was seated comfortably on a chair without armrests. The shoulder was adducted and neutrally rotated, with the elbow at 90° flexion, and the forearm and wrist in a neutral position (20). Measurements started with the dominant hand. Three measurements (in kilograms) were obtained at 15 s intervals and the mean value was analyzed.

### Ethical Approval

The study was approved by the Medical Research Ethics Committee Ministry of Health Malaysia [NMRR-17-3489-38159 (IIR)].

### Statistical Analysis

Data analysis, for descriptive and multivariate analysis were performed using SPSS software (Version 21; SPSS Inc., Chicago, Illinois). Baseline descriptive statistics were presented in the form of mean (standard deviation) and frequency (percentage) for continuous and categorical variables, respectively. To determine the potential confounder in the study, baseline comparisons were done for the outcome variables namely timed up and go, Berg balance, sit-to-stand and hand grip strength, with sociodemographic and morbidity variables. Pearson correlation test was used to test potential association when both test variables were continuous. Independent *t*-test was used to compare study variables between two groups while one-way Analysis of Variance (ANOVA) was applied for more than two groups. For the pre- and post-intervention evaluations, paired *t*-test was used to examine the significant difference between post- and pre-intervention. All significance was set at 0.05. In addition, Cohen's effect size (ES) was used to evaluate the impact of the intervention on the outcome variables (21). Cohen's ES either measured the sizes of associations between variables or the sizes of differences between means. Cohen's effect size in this study was calculated by subtracting the mean of post-intervention to pre-intervention and the result was divided by pooled standard deviation. Cohen suggested that effect size <0.10 would be considered a “trivial” effect size, 0.1–0.3 represented a “small” effect size, 0.3–0.5 as “moderate” and more than 0.5 a “large” effect size.

## Results

A total of 32 older persons aged 60 years or older with pre-frail symptoms were recruited from the community center. From a total of 36 participants who began the study at the baseline, 32 participants remained until the end of the study corresponding to 88.9% of adherence rate. The dropout cases were removed from the analysis. The mean age of the respondents was 66.8 ± 4.76SD. More than half (56.3%) of the participants were male. Approximately 47% of respondents had received secondary education followed by primary (37.5%) and tertiary (15.6%). A majority of them were married (93.8%) and never smoked (77.0%). As for comorbidity status, 21.9% had no disease, 37.5% had at least one disease, 15.6% presented with two diseases and 25.0% were burdened with up to three diseases. Participant characteristics is presented in Table 2.

**Table 2:** Characteristics of subjects and baseline comparison for timed up and go, Berg balance, sit-to-stand, and hand grip strength measurement.

**Study variable**	**Number (%)**	**Mean** **±** **SD**			
		**TUG**	**BB**	**STS**	**HG**
Age	66.81 ± 4.76^a^	0.10^b^	−0.18^b^	0.13^b^	0.14^b^
**Gender**
Male	14 (43.7)	12.03 ± 3.82	52.14 ± 6.31	13.91 ± 2.88	26.21 ± 3.77^*^
Female	18 (56.3)	10.62 ± 2.28	53.17 ± 2.41	14.57 ± 3.13	15.33 ± 4.84^*^
**Education level**
Primary	12 (37.5)	10.61 ± 3.33	52.08 ± 3.00	13.72 ± 3.95	20.54 ± 7.73
Secondary	15 (46.9)	11.73 ± 3.20	52.53 ± 5.89	14.92 ± 2.25	19.67 ± 5.93
Tertiary	5 (15.6)	11.31 ± 2.28	54.80 ± 1.79	13.76 ± 2.40	20.30 ± 9.50
**Marital status**
Single	2 (6.2)	8.89 ± 0.70	53.50 ± 2.12	12.83 ± 0.09	11.25 ± 3.89
Married	30 (93.8)	11.40 ± 3.12	52.67 ± 4.62	14.38 ± 2.99	20.68 ± 6.78
**Smoking status**
Never smoked	26 (77.0)	11.12 ± 2.66	53.35 ± 2.50	14.41 ± 2.88	18.62 ± 6.92
Ever smoked	6 (23.0)	11.79 ± 4.81	50.00 ± 9.10	13.74 ± 3.68	26.50 ± 1.64
**Comorbidity**
0	7 (21.9)	0.07^b^	−0.37^b^*^^	0.03^b^	0.11^b^
1	12 (37.5)				
2	5 (15.6)				
3	8 (25.0)				

*%, Percentage; TUG, Timed up and go; BB, Berg balance; STS, Sit-to-stand; HG, Hand grip strength*.

*Mean comparison between groups was done using independent t-test for gender, marital status and smoking status while ANOVA was used for education level. Pearson correlation test was used for numerical variables; age and comorbidity*.

a *Mean ± Standard deviation*.

b *Pearson correlation coefficient*.

* *p ≤ 0.05*.

Table 2 shows baseline comparison of study outcome variables with sociodemographic and comorbidity variables. The results of the univariate analysis of Pearson's correlation, independent *t*-test and one-way ANOVA for the related variables, respectively, showed that the mean of the hand grip strength was significantly different among male and female participants. Comorbidity number was also significantly associated with Berg balance.

The results of the paired *t*-test (Table 3) revealed non-significant *p*-value for pre- and post-intervention comparison for all study outcomes; timed up and go (*t* = −0.78, *p*-value = 0.438), Berg balance (*t* = −0.03, *p*-value = 0.957), sit-to-stand (*t* = −1.11, *p*-value = 0.274) and handgrip strength (*t* = 0.87, *p*-value = 0.391). However, observing the values of mean difference, the study intervention was shown to have slightly improved the time up and go (Mean difference = −0.25), and sit-to-stand duration (Mean difference = −0.41) as well as the handgrip strength (Mean difference = 0.68) among the participants. On the assessment of Cohen ES, all three improvements exhibited small effect size. Sit-to-stand duration was shown to have most benefited from the intervention with highest ES among the outcome variables (ES = 0.20).

**Table 3:** Pre- and post-intervention comparisons using paired t-test for timed up and go, Berg balance, sit-to-stand, and hand grip strength measurement.

**Study variable**	**Mean** **±** **SD**		**Mean Diff. (95% CI)**	**Percentage of improvement, *n* (%)**	***t* (df)**	***p*-value**	**ES**
	**Pre**	**Post**					
TUG	11.24 (3.08)	10.99 (3.06)	−0.25 (−0.89, 0.39)	13 (40.6)	−0.78 (31)	0.438	0.14
BB	52.71 (4.49)	52.68 (4.78)	−0.03 (−1.21, 1.15)	12 (37.5)	−0.05 (31)	0.957	–
STS	14.29 (2.99)	13.87 (13.87)	−0.41 (−1.17, 0.34)	16 (50.0)	−1.11 (31)	0.274	0.20
HG	20.09 (6.99)	20.78 (6.40)	0.68 (−0.92, 2.30)	17 (53.1)	0.87 (31)	0.391	0.15

*SD, Standard deviation; Mean Diff., Mean difference; t, t-value; df, degree of freedom; ES, Cohen's effect size; TUG, Timed up and go; BB, Berg balance; STS, Sit-to-stand; HG, Hand grip strength*.

*Paired t-test was conducted separately for each study variable as outcome of the study*.

## Discussions

Baseline data showed there is significant difference of mean hand grip strength between men (26.21 ± 3.77 kg) and women (15.33 ± 4.84 kg). An agreement was found with another study where the determinants of hand grip strength were reported to include gender and age effect (16). However, the mean hand grip strength in the present study was lower as compared to other populations such as Europeans (41.26 kg for men, 24.87 kg for women), Japanese-American men (36.65 kg) and South African (37.9 kg for men, 31.5 kg for women) (22–24). The present study also indicates that the number of co-morbidity was correlated with the Berg balance score. About 41% of the participants reported to have more than one morbidity, which could increase risk of falls among the pre-frail older persons.

This study aimed to evaluate the effects of COME intervention program on upper and lower extremity muscle strength. Loss of muscle mass and decreased muscle strength among the frail older persons resulted in functional impairment and increased risk of falling. The data show a tendency toward improvements with no significant improvement. Sit-to-stand was shown to have most benefited from the intervention indicated improvement on lower extremities muscle strength. Other studies exhibited similar effects of a variety of approaches of strength training such as resistance and vibration to the upper and lower extremity strength (25–27). The improvements of muscle strength may be explained by the capacity of skeletal muscles to increase its satellite cells proportion (28) and capillarization (29) through physical activity. Even though the ability of skeletal muscle to regenerate is compromised with aging, it retains the ability to positively respond to stimuli, such as exercise (30). Therefore, exercise targeted upper and lower extremities muscles could delay or prevent pre-frail older persons to become frail.

On the other hand, this exercise intervention is not effective in improving balance among pre-frail elderly. As the current study was targeted to strengthen upper and lower extremity muscles, the exercise movements targeted major muscles in upper (shoulders, upper arms, back, chest and abdomen) and lower extremities (hips, thighs, knees, lower legs and ankle), and was conducted in standing or sitting position. Researchers stressed out that the importance of training balance should consider complex motor behavior with more challenging movements than linear walking, such as walking-and-turning, where the turn-related changes in feet, trunk, and head movements are integral part of the kinematics of steering a body (31, 32). Thus, post-intervention Berg balance score of the current study showed no improvement in balance among the participants.

Despite, Distefano and Goodpaster concluded from their study that age-related loss of muscle strength and regenerative capacity could not be completely prevented by just a single intervention or training (33). However, constant exercise can significantly reduce, or prevent the declines in muscle metabolism and function due to the ability of skeletal muscle to retain its plasticity to some extant in response to exercise, thus providing convincing evidence that many of the negative age-related changes in muscle function and metabolism are caused by sedentary lifestyle secondary to aging.

## Conclusion

COME intervention program showed favorable trend toward improvement of upper and lower extremities muscle strength. This study should be further tested in randomized control trial to confirm its effectiveness. Moreover, this pilot study demonstrated the feasibility of community-based exercise program and its potential to increase muscle strength among pre-frail older persons.

## Data Availability Statement

The raw data supporting the conclusions of this article will be made available by the authors, without undue reservation.

## Ethics Statement

The studies involving human participants were reviewed and approved by Medical Research Ethics Committee Ministry of Health Malaysia. The patients/participants provided their written informed consent to participate in this study.

## Author Contributions

RR and HMi conceived the study and participated in the experimental design, protocol development, and drafted the manuscript. HMa participated in formal analysis of data and drafted the manuscript. AA participated in experimental design and drafted the manuscript. All authors contributed to the article and approved the submitted version.

## Conflict of Interest

The authors declare that the research was conducted in the absence of any commercial or financial relationships that could be construed as a potential conflict of interest.
